# Predicting Affect Classification in Mental Status Examination Using Machine Learning Face Action Recognition System: A Pilot Study in Schizophrenia Patients

**DOI:** 10.3389/fpsyt.2019.00288

**Published:** 2019-05-06

**Authors:** Ran Barzilay, Nadav Israel, Amir Krivoy, Roi Sagy, Shiri Kamhi-Nesher, Oren Loebstein, Lior Wolf, Gal Shoval

**Affiliations:** ^1^Geha Mental Health Center, Petach Tikva, Israel; ^2^Sackler School of Medicine, Tel Aviv University, Tel Aviv, Israel; ^3^Lifespan Brain Institute, Department of Child and Adolescent Psychiatry and Behavioral Sciences, Children’s Hospital of Philadelphia and Penn Medicine, Philadelphia, PA, United States; ^4^The Blavatnik School of Computer Science, Tel Aviv University, Tel Aviv, Israel

**Keywords:** affect, face recognition, machine learning, schizophrenia, clinical psychiatry

## Abstract

Classifying patients’ affect is a pivotal part of the mental status examination. However, this common practice is often widely inconsistent between raters. Recent advances in the field of Facial Action Recognition (FAR) have enabled the development of tools that can act to identify facial expressions from videos. In this study, we aimed to explore the potential of using machine learning techniques on FAR features extracted from videotaped semi-structured psychiatric interviews of 25 male schizophrenia inpatients (mean age 41.2 years, STD = 11.4). Five senior psychiatrists rated patients’ affect based on the videos. Then, a novel computer vision algorithm and a machine learning method were used to predict affect classification based on each psychiatrist affect rating. The algorithm is shown to have a significant predictive power for each of the human raters. We also found that the eyes facial area contributed the most to the psychiatrists’ evaluation of the patients’ affect. This study serves as a proof-of-concept for the potential of using the machine learning FAR system as a clinician-supporting tool, in an attempt to improve the consistency and reliability of mental status examination.

## Introduction

The determination of mood and affect is a major aspect of the psychiatric mental status examination (MSE) (1, 2). However, in contrast to other aspects of the mental status, mood and affect determination is often challenging for clinicians, especially during the initial part of their careers (3–6). Although definitions of the terms mood and affect often vary, mood is typically viewed as referring to the patient’s internal, subjective, and more sustained emotional state, whereas affect relates to the patient’s externally observable and more changeable emotional state (1). Few studies to date have described the inconsistent apprehension of what is mood and affect between clinicians (5–7). The inconsistency in the way different clinicians understand the concept of mood and affect consequently contributes to differences in the identification of affect between clinicians (8). This finding underscores the need to develop reliable tools that may serve as an aid to clinicians when conducting a psychiatric mental status examination, a concept that was presented long ago (9, 10), but is gaining interest as science advances and as machine learning discipline shows the capacity of developing decision supporting tools for psychiatrists (11).

Computer face recognition systems allow identification of a person from a digital image or a video frame. Though it has been mostly used in the context of security and surveillance, the applications of such technology are far more diverse (12). In medicine, applications of facial recognition include the identification of rare genetic disorders that result in unique facial features (13, 14).

An even newer field is Face Action Recognition (FAR), a research domain that has made great strides over the past decade (15). In some real-world applications, the goal is to recognize or infer intention or other psychological states rather than facial actions alone. There are many applications in the FAR field, including human-computer interfaces, video surveillance and patient condition monitoring. A FAR system is normally composed of four main steps: (i) face detection and tracking, (ii) face alignment-which is based on locating semantic facial landmarks such as the eyes, nose, mouth and chin (iii) feature extraction, and (iv) classification. While steps (i)—(iii) are done with computer vision techniques, step (iv) is done with machine learning algorithms such as the Support Vectors Machine (SVM) algorithm.

In the current study, we applied FAR machine learning algorithms to classify affects based on the classification of five psychiatrists that rated the affects of 25 male schizophrenia patients that underwent a videotaped semi-structured psychiatric interview. We aimed to achieve two goals: (1) to show that in a clinically homogenous patient population, the rating of affect is highly heterogenous among experienced clinicians and (2) to employ a unique, novel FAR system that we had previously developed (16) in an attempt to identify affect in the MSE, which may in turn lead to harmonization and better standardization of the MSE.

## Methods and Procedure

### Study Population

All patients were males diagnosed with schizophrenia (n = 25, age 18-70), hospitalized at Geha Mental Health Center. All patients were diagnosed by at least two senior psychiatrists according to DSM IV-TR criteria (17). The psychiatrists (n = 5) who rated the videotaped interviews were all senior specialists in general adult psychiatry, each with at least 10 years of experience. The investigation was carried out in accordance with the latest version of the Declaration of Helsinki and the study design was reviewed and approved by the institutional review board. Written informed consent of the participants was obtained after the nature of the procedures had been fully explained. Demographic data of study subjects was obtained from the medical records and can be found in **Supplemental Data**.

### Psychiatric Interview

All interviews were videotaped by the interviewer using a high definition digital video camera recorder (Sony HDR-XR500, Japan). All interviews lasted between 7 and 10 minutes. All patients were interviewed by the lead author (RB), a resident psychiatrist, in a semi-structured interview composed of the following 10 questions: (1) Can you please present yourself and tell me a bit about yourself? (2) How do you feel? (3) Can you tell me about the events that led to your current hospitalization? (4) Can you tell me some things about your family? (5) Can you tell me of something sad that has recently happened to you? (6) Can you tell me of something pleasing that has recently happened to you? (7) Is there anything else you want to add? (8) What do you think about the recent situation in the country? (9) What are your future plans? (10) How did you feel about talking with me in front of the camera?

### Measures of Affect

Annotation of videos was performed for three domains of affect, as described in the psychiatric MSE chapter of a classic textbook of Psychiatry (2). **Quality** of affect was annotated as either dysphoric, euthymic or manic. **Range** of affect was classified as full, restricted, flat or blunt. **Subtype** of affect was categorized as one of the twelve: stupid, euphoric, empathetic, self-contemptuous, anxious, suspicious, hopeless, frightened, irritable, with a sense of guilt or other (2).

### Face Recognition Procedures

**Figure 1:**
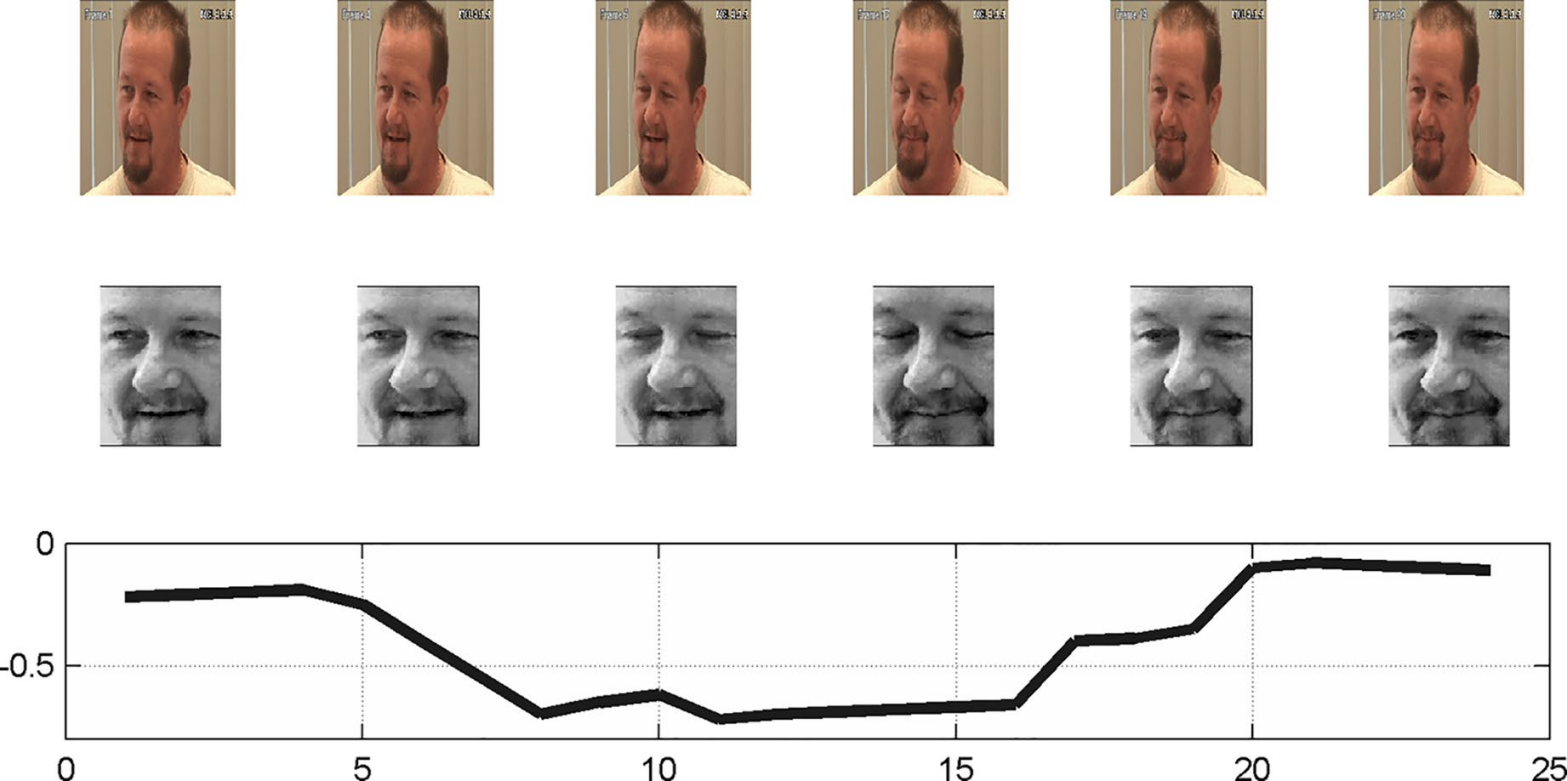
Generation of a normalized face image. (Top) The input frames (1, 4, 5, 17–19); (Middle) Normalized face; (Bottom) The mean gray level intensity of left eye cell along time domain. Image was taken from painDB dataset (20), a publically available data source. The person in the figure is not related to the study.

**Figure 2:**
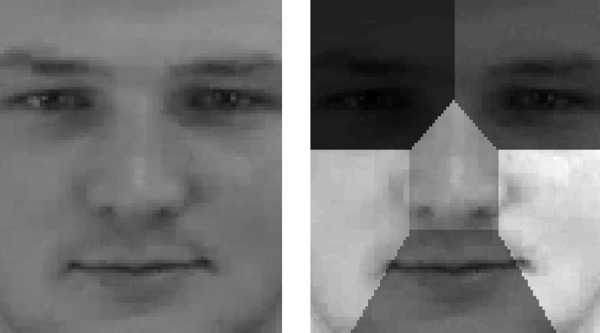
(Left) Normalized face. (Right) The face divided to six parts: left and right eye, left and right cheek, mouth and nose. Image was taken from FEED database (22), a publically available data source. The person in the figure is not related to the study.

The quality of the extracted features representing the video content plays a key role for the classification task that is required for the affect classification. A detailed description of the image processing and computational complexity can be found in our previous report (16). In brief, the proposed system is designed to be reliable and rapid and support real-time analysis applications. The method is based on capturing local changes and encoding these local motions into a histogram of frequencies. In this approach, a face video is modeled as a sequence of histograms by the following procedure: (1) An input face image aligned and is cropped to generate a normalized face image (**Figure 1**); (2) Each normalized frame is divided into a grid of equally sized cells; (3) The mean gray level intensity of each cell *r* at frame *n* is recorded as *X* *_r_*[*n*].

For the analysis of which face part contributes the most for the affect annotation, we divided the facial grid to six parts: left and right eye, left and right cheek, mouth and nose (**Figure 2**).

#### Mid-Level Features

The labels employed in psychiatry to capture the facial appearance attain to multiple aspects of the face’s dynamics. We therefore devised three mid-level features, which capture the facial expression scores, the dominating expression labels, and the facial motion. These vectors have previously been described (16). Briefly: (1) Expression - for each frame in a video, we predicted the score of seven expressions (norm, anger, disgust, fear, happiness, sadness and surprise), by employing a classifier that is based on the recorded mean intensities, denoted by *X_r_*[*n*] above. In order to describe a video, we aggregated the expression scores to one vector of length 7 that describes a video. (2) Label - we calculated the variability of the expressions in a video, by predicting the dominating expression for each frame and then calculating the number of transitions of the label in a video. (3) Motion – calculated by taking the standard deviation of gray level (average the gray level for each frame in a video) of the face. This calculation simply captures the variance of the face in a video.

In addition, since in some cases a label might be more evident in one vector type than in another, we also consider the combination of the different vectors. These are obtained by concatenating (4) Motion and label, or (5) Motion and expression.

#### Classifiers

A linear support vector machine (SVM) was used as the classifier for the proposed method. Specifically, we use the implementation of LIBSVM (21). The basic SVM method outputs binary decisions, and the multiclass classification required for our application (three classes for quality; four classes for range; twelve classes for subtype) is accomplished by using the one-versus-all rule. During training, a classifier is learned to separate each class from the rest. For example, for the quality classifier, a first binary classifier treats dysphoric cases as positive cases, and euthymic or manic cases as negative cases, a second classifier labels only euthymic cases as positive, the rest as negative, and a third classifier labels only manic cases as positive. Given a sample to classify, each classifier outputs a score that is aimed to be negative if the sample is from the negative group, and positive for a sample that is associated with the positive label. Once training is complete, a test video is assigned to the label of the binary classifier with the highest response.

In our experiments, the classifiers are employed as part of a Leave One Out procedure, as explained in the next section.

### Statistical Methods

We perform statistical analysis in order to assess the following: (i) the agreement between the five raters, (ii) the predictive power of the automatic method, (iii) the predictive power per facial part, and (iv) the success of the machine learning procedure in predicting the rating of each rater.

For measuring the agreement between a pair of raters, we consider the ratings given by the two over all cases. We then record the ratio of the cases in which the two provided the same annotation.

In order to measure the predictive power of each one of the three types of vectors (and two combinations thereof), we employ a logistic regression analysis. This is done separately for each rater and to each vector type (the five types described in Sec. 2.4.1) and is repeated to each domain: quality, range, and subtype of affect. Each separate analysis (a total of 5 types of vectors, including the 2 hybrid types, times 5 raters, times 3 domains, or a total of 75 experiments) employs the classes of the domain as dependent variables. For example, for the 25 experiments (5 vector types times 5 raters) that involve the affect’s quality, there are three classes in the regression problem: Dysphoric, Euthymic and Manic.

In each experiment, we perform multinomial logistic regression using the R (version 0.99.902) package mlogit (version 0.2-4). The p-value reflects the Likelihood ratio test vs. a baseline model, which does not observe the features (only the distribution of the labels).

In order to compare the predictive power of each facial part separately, these experiments are then repeated for the Motion descriptor only (the third feature vector in Sec. 2.4.1) for each of the six facial regions for the three domains. The other features require the classification of the facial expression, which in turn is based on the entire face. Here, too, the p-values are obtained using multinomial logistic regression.

While the multinomial regression experiments provide statistical significance, we employ Support Vector Machines (SVM) in order to estimate the classification accuracy, i.e., to measure the success in predicting each rater. This is done to each rater separately, due to the lack of consensus. A Leave One Out procedure (LOO) is used, in which each patient provides the test sample once. The samples of all other patients are taken as the training samples and the learned classifier is used to predict the target label of the held-out sample. This is repeated to all patients.

For each test, we considered p-value < 0.05 to represent statistical significance.

## Results

### Agreement between Raters

We first evaluated the agreement between the five psychiatrist raters. It is immediately evident that the distributions of each of the labels vary considerably between the raters, in all three domains of affect investigated in this study: quality (**Table 1A**), range (**Table 1C**) and subtype of affect (**Table 1E**).

**Table 1A:** Distribution of affect quality classification by the five psychiatrists.

Psychiatrist	Dysphoric	Euthymic	Manic
**Rater 1**	0%	100%	0%
**Rater 2**	12.5%	70.8%	16.6%
**Rater 3**	54.1%	41.6%	4.17%
**Rater 4**	4.16%	87.5%	8.33%
**Rater 5**	8.33%	91.66%	0%

**Table 1C:** Distribution of affect range classification by the five psychiatrists.

Psychiatrist	Full	Restricted	Blunt	Flat
**Rater 1**	4.1%	25%	50%	20.8%
**Rater 2**	0%	12.5%	66.6%	20.8%
**Rater 3**	16.6%	41.6%	25%	16.6%
**Rater 4**	8.33%	50%	37.5%	4.16%
**Rater 5**	4.16%	12.5%	54.16%	29.16%

**Table 1E:** Distribution of affect sub-type classification by the 5 psychiatrists.

	Rater 1	Rater 2	Rater 3	Rater 4	Rater 5
**Unknown**	21%	33%	0%	50%	88%
**Stupid**	54%	13%	17%	8%	8%
**Euphoria**	4%	8%	8%	4%	0%
**Empathetic**	8%	0%	8%	4%	0%
**Self contempt**	0%	0%	0%	0%	0%
**Anxious**	13%	4%	17%	17%	0%
**Suspicious**	0%	13%	4%	0%	0%
**Hopeless**	0%	4%	0%	8%	0%
**Frightened**	0%	0%	13%	0%	0%
**Irritable**	0%	13%	8%	4%	0%
**Vacancy**	0%	13%	25%	4%	4%
**Sense of guilt**	0%	0%	0%	0%	0%

The agreement between the raters was defined as the number of identical annotations between two raters divided by the number of samples. We found that there was a moderate agreement between the raters when annotating the quality of the affect (54% ± 20, mean ± SD, **Table 1B**) that declined when annotating the range of affect (43% ± 16, mean ± SD, **Table 1D**). The agreement is even lower when annotating the specific subtype of the affect, where there are more options (25% ± 13, mean ± SD ,**Table 1F**).

**Table 1B:** Agreement between the five psychiatrists in the classification of quality of affect. Values represents percentage of agreements of specific affect subtype classification. It is calculated as the number of “affects” that two psychiatrists agree divided by the number of all videos samples and multiplied by 100.

Psychiatrist	Rater 1	Rater 2	Rater 3	Rater 4	Rater 5
**Rater 1**	100%	71%	42%	88%	92%
**Rater 2**	71%	100%	46%	58%	63%
**Rater 3**	42%	46%	100%	38%	33%
**Rater 4**	88%	58%	38%	100%	88%
**Rater 5**	92%	63%	33%	88%	100%

**Table 1D:** Agreement between the five psychiatrists in the classification of range of affect. Values represents percentage of agreements of specific affect subtype classification, which are calculated as the number of “affects” that two psychiatrists agree on divided by the number of all videos samples, and multiplied by 100.

Psychiatrist	Rater 1	Rater 2	Rater 3	Rater 4	Rater 5
**Rater 1**	100%	58%	38%	38%	58%
**Rater 2**	58%	100%	25%	33%	75%
**Rater 3**	38%	25%	100%	42%	33%
**Rater 4**	38%	33%	42%	100%	29%
**Rater 5**	58%	75%	33%	29%	100%

**Table 1F:** Agreement between the five psychiatrists in the classification of subtype of affect. Values represents percentage of agreements of specific affect subtype classification. The values are calculated as the number of “affects” that two psychiatrists agree on divided by the number of all videos samples, multiplied by 100.

Psychiatrist	Rater 1	Rater 2	Rater 3	Rater 4	Rater 5
**Rater 1**	100%	17%	25%	17%	21%
**Rater 2**	17%	100%	29%	29%	38%
**Rater 3**	25%	29%	100%	21%	0%
**Rater 4**	17%	29%	21%	100%	50%
**Rater 5**	21%	38%	0%	50%	100%

### Identification of Features That Enable Affect Annotation

We employ five types of mid-level features, as described in Sec. 2.4.1, in order to capture various aspects of the face in a clinical setting. To evaluate the relevance of these vectors to each rater’s annotation capacity, the vectors were put in a regression analysis, with the quality, range or sub-type of affect as the dependent variable. This process comprises the machine learning capacity of the algorithm and allows the prediction of each rater’s annotation given a new video.

Logistic regression analyses P-values for each classifier for the affect quality, range and subtype are detailed in **Table 2A–C**. As can be seen, the quality dimension is captured in a statistically significant way, for most raters, using the motion feature vector. The hybrid motion+label based classifier is highly predictive of the range labeling in at least four out of five raters. The expression feature is significantly associated with subtype in at least four out of five raters.

**Table 2:** Values represents the P-values of logistic regression analyses for each classifier for the affect (A) quality, (B) range and (C) subtype. P-values < 0.05 are marked in bold. Psychiatrist 1 is missing due to lack of variance in their quality labeling.

(A) Quality					
Psychiatrist	Expression	Motion	Label	Motion+label	Motion+expression
**Rater 1**					
**Rater 2**	0.09	**0.03**	0.15	0.07	0.05
**Rater 3**	**0.01**	0.33	**0.00**	0.33	0.11
**Rater 4**	0.11	**0.04**	0.18	0.08	0.69
**Rater 5**	0.17	**0.03**	0.85	0.06	0.39
(B) Range					
Psychiatrist	Expression	Motion	Label	Motion+label	Motion+expression
**Rater 1**	**0**	0.32	**0**	0.06	**0.04**
**Rater 2**	0.08	**0.01**	0.65	**0.01**	**0.03**
**Rater 3**	0.53	**0**	0.59	**0.00**	**0.01**
**Rater 4**	0.18	**0.02**	**0.04**	**0.01**	0.10
**Rater 5**	0.05	0.55	**0.01**	**0.01**	0.08
(C) Subtype					
Psychiatrist	Expression	Motion	Label	Motion+label	Motion+expression
Rater 1	0	0.10	0.69	0.60	0.40
Rater 2	0	0.00	0.45	0.00	0.51
Rater 3	0	0.21	0.17	0.00	0.42
Rater 4	0	0.00	0.10	0.00	0.62
Rater 5	0.7	0.04	0.43	0.08	0.69

### Identification of Face Parts That Are Used by Raters to Enable Affect Annotation

We then proceeded to investigate which parts of the face are being used by the psychiatrist to determine the affect annotation for quality, range and sub-type. To that end, we divided the face of the subjects to six parts: left and right eye, left and right cheek, mouth and nose (**Figure 2**), and analyzed the classifier based on the motion features for each rater to determine the relative contribution of each face part to the annotation in each rater. According to the tables below, describing the measured p-values across all annotations, we found that the eyes are mostly associated with the psychiatrists’ decision regarding affect annotation. Our conclusions are derived from the number of p-values below 5% from all raters. **Tables 3** summarize the results of the Logistic regressions for face parts contribution to the affect quality (**Table 3A**), range (**Table 3B**) and subtype (**Table 3C**).

**Table 3:** Regression analysis of the contribution of each face part to the classification of (A) quality, (B) range and (C) subtype of affect. Values represent P-values calculated for each face part. Significant P-values < 0.05 are marked in bold.

(A)										
Psychiatrist	Eyes	Mouth and nose	Cheeks	All parts	Left eye	Right eye	Nose	Mouth	Left cheek	Right cheek
**Rater 1**										
**Rater 2**	**0.04**	0.13	0.63	0.11	**0.03**	0.20	0.07	0.46	0.44	0.28
**Rater 3**	0.28	0.47	0.33	0.23	0.09	0.08	0.41	0.18	0.32	0.13
**Rater 4**	**0.00**	**0.00**	**0.00**	**0.01**	0.05	0.19	**0.03**	0.38	**0.04**	**0.01**
**Rater 5**	**0.00**	**0.00**	**0.00**	**0.00**	**0.02**	0.17	**0.02**	0.21	**0.01**	0.29
(B)										
Psychiatrist	Eyes	Mouth and nose	Cheeks	All parts	Left eye	Right eye	Nose	Mouth	Left cheek	Right cheek
**Rater 1**	0.50	0.23	0.39	0.26	0.65	0.45	0.52	0.08	0.32	0.39
**Rater 2**	0.06	0.07	0.42	0.29	0.10	0.06	0.15	0.10	0.16	0.89
**Rater 3**	**0.04**	0.22	0.35	0.35	0.05	0.07	0.10	0.17	0.69	0.18
**Rater 4**	0.30	0.21	0.14	0.43	0.34	0.35	0.39	0.30	0.25	0.05
**Rater 5**	0.42	0.05	0.84	0.38	0.52	0.19	0.12	0.29	0.63	0.80
(C)										
Psychiatrist	Eyes	Mouth and nose	Cheeks	All parts	Left eye	Right eye	Nose	Mouth	Left cheek	Right cheek
**Rater 1**	0.57	0.06	0.51	0.44	0.89	0.53	0.16	0.12	0.56	0.71
**Rater 2**	0.05	0.13	0.16	0.34	0.50	0.08	0.50	0.59	0.22	0.17
**Rater 3**	0.69	**0.04**	0.35	0.77	0.86	0.79	0.37	0.31	0.09	0.70
**Rater 4**	**0.01**	0.13	0.01	0.71	0.02	0.06	0.22	0.39	0.38	0.01
**Rater 5**	0.26	0.27	0.05	0.17	0.79	0.10	0.89	0.16	0.36	0.17

### Utilization of The Machine Learning Algorithms to Predict Each Rater’s Affect Annotation

Finally, we explored the potential of the machine learning algorithms to predict each rater’s annotation of the quality (**Table 4A**) and the range (**Table 4B**) of the affect. Using the features described previously and the SVM classifier, we have predicted the annotation of raters for each patient. This was done in a LOO (Leave-one-out) manner, in order not to make predictions on the training set. Since the distribution of the labels is not uniform, the chance level ranges between the various raters, and we therefore also report the improvement over baseline calculated as the ratio between the obtained accuracy and the frequency of the most common label for each rater^1^.

**Table 4:** Results of the Machine Learning classifier of each rater for Quality (A) and Range (B). Shown are both the obtained accuracies and the improvement coefficient computed as the ratio between the obtained accuracy and the accuracy obtained by guessing the most common label. Rater 1 did not have variability in Quality and therefore this row is removed from panel A.

A – SVM classifier Quality prediction		
Psychiatrist	Accuracy	Improvement
**Rater 1**		
**Rater 2**	75.0%	1.17
**Rater 3**	79.1%	1.46
**Rater 4**	91.6%	1.05
**Rater 5**	91.6%	1
B – SVM classifier Range prediction		
Psychiatrist	Accuracy	Improvement
**Rater 1**	75.0%	1.5
**Rater 2**	75.0%	1.13
**Rater 3**	50.0%	1.2
**Rater 4**	70.8%	1.41
**Rater 5**	70.8%	1.3

## Discussion

In this study, we show that the use of a computerized face action recognition system may be used to predict the identification of affect by psychiatrists in real world schizophrenia patients. The first finding of our study was that even in the relatively homogenous population of hospitalized male schizophrenia patients, there is a lack of inter-rater reliability between five senior adult psychiatrists working in the same mental health center. This finding is often assumed in the clinical psychiatric setting, but we found no data on that in the literature.

The second finding is that automatic facial analysis may be able to predict the label provided by psychiatrists. This finding is of interest as it may suggest a possibility to develop a consistent clinician-supporting tool that may add a standardized aspect to the mental status examination, consequently contributing to increased consistency of clinical diagnoses (18). Developing standardized objective measures remains a major challenge in the field of psychiatry, as inter-rater reliability is currently low, and no gold-standard ‘valid’ measure is available to evaluate the profound disturbances observed in the affects of schizophrenia patients as well as other psychiatric patients. Noteworthy, better performance is expected in subjects without diminished facial expression, such as patients with depression or anxiety disorders. This remains to be tested in future studies.

A possible future implication of such a system may be as part of a psychiatrist-supporting tool to assist in clinical decision-making based on future studies exploring the correlations of the system’s output with clinical variables, such as severity of psychosis, negative symptoms and prognosis.

Another potential implication could be predicting the risk and timing of onset of schizophrenia in high-risk patients in the prodromal phases of the disorder. Here, tracking the progress of the patient’s affect over time could be of a major interest.

In addition, evaluating the facial affects of patients using this system in structural and functional neuroimaging studies may reveal endophenotypic correlates, that may shed light on the mechanisms underlying affect disturbances in schizophrenia.

Our study has some limitations, most prominent is the sample size of 25 interviews and 5 psychiatrist raters from the same hospital. However, we aimed at providing a proof of concept for the utility of a face action recognition system in clinical psychiatry, and the fact that our method is able to predict the affect classification despite inter-rater divergence acts to show that there may be a place for face action recognition systems in clinical psychiatry. Another limitation that might be suspected is the fact that the psychiatrists based their rating on a 10-minute standardized interview, while a full mental status evaluation is often based on a longer interview. The homogenous subject population in our study was confined to male schizophrenia inpatients, making it hard to deduce from the current findings to other clinical populations. We chose to conduct our pilot study on a population that is known to have restricted affect (19, 23), with the hope that this population will enable maximum chances to show feasibility due to expected consistency among raters. Importantly, since schizophrenia patients are characterized by restricted-range affect, we suggest that the fact that we managed to show efficacy of the face action recognition tool in such a homogenous population rather acts to strengthen our findings. As the general population of psychiatric patients is more diverse in terms of affect, it is fair to assume that there will be even lower inter-rater consistency between clinicians, making the tool we present in this study even more useful. Future studies should implement the approach we propose in populations with more salient affective features such as mood disorders. Another limitation of our study is that we did not have Brief Psychiatric Rating Scale (BRPS) or Positive and Negative Syndrome Scale (PANSS) measures for the participants.

To conclude, we provide proof of concept for the use of a face action recognition system in the classification of affect, a key part of the mental status examination that is known for poor inter rater consistency. Though preliminary, the results may pave a way for the future use of face action recognition technologies as a clinician-supporting tool in real-life clinical psychiatry. Future studies should aim to explore such approach in wider clinical populations and for other clinical implications.

## Ethics Statement

The investigation was carried out in accordance with the latest version of the Declaration of Helsinki and the study design was reviewed and approved by the institutional review board of Geha Mental Health Center. Written informed consent of the participants was obtained after the nature of the procedures had been fully explained.

## Author Contributions

RB designed the study, was the coordinator of the study and wrote the first draft of the manuscript. NI designed the study, developed the algorithm and managed the statistical analysis. AK, RS, SK and OL took part in the methodological procedure (rated the patients' affect in all videos). LW designed the study, developed the algorithm and managed the statistical analysis. GS designed the study, wrote the protocol and took part in the methodological procedure (rated the patients' affect in all videos). All authors contributed and have approved the final version of the manuscript.

## Conflict of Interest Statement

The authors declare that the research was conducted in the absence of any commercial or financial relationships that could be construed as a potential conflict of interest.

## Footnotes

1 Without looking at the input, guessing the most common label is the optimal strategy. It is considerably better for the uneven distribution of labels encountered here than predicting uniformly among the classes, and is also better than picking a label according to the distribution of the labels of the samples observed in training.
