# Supplementary material for: Predicting Affect Classification in Mental Status Examination Using Machine Learning Face Action Recognition System: A Pilot Study in Schizophrenia Patients
Source: Front Psychiatry. 2019 May 6;10:288. doi: 10.3389/fpsyt.2019.00288 (PMC6512891; doi:10.3389/fpsyt.2019.00288)
Supplement: Supplementary file 1 [file Table_1.docx]

Supplemental data 1- Demographic data of patients with schizophrenia

|  | Age (years) | Illness  duration | Antipsychotic Medications | Comorbidities |
| --- | --- | --- | --- | --- |
| #1 | 26 | 2 | Atypical | Affective symptoms |
| #2 | 36 | 6 | Typical | Anti-Social Personality Disorder, Drug use (cannabinoids) |
| #3 | 36 | 1 | Typical and Atypical |  |
| #4 | 43 | 10 | Typical | Gambling Disorder |
| #5 | 40 | 16 | Typical | Panic Disorder |
| #6 | 33 | 6 | Typical and Atypical | Drug use (stimulants) |
| #7 | 43 | 13 | Atypical |  |
| #8 | 47 | 30 | Typical | Obsessive Compulsive Disorder |
| #9 | 51 | 19 | Atypical |  |
| #10 | 24 | 5 | Atypical | Affective symptoms |
| #11 | 38 | 15 | Typical | Affective symptoms |
| #12 | 49 | 22 | Typical and Atypical |  |
| #13 | 34 | 2 | Typical |  |
| #14 | 29 | 7 | Atypical | Poly drug use, Alcohol |
| #15 | 52 | 30 | Typical |  |
| #16 | 44 | 20 | Typical and Atypical | Major Depression, Alcohol |
| #17 | 25 | 1 | Atypical |  |
| #18 | 38 | 19 | Typical and Atypical | Affective symptoms |
| #19 | 43 | 16 | Atypical | Panic Disorder |
| #20 | 48 | 31 | Atypical |  |
| #21 | 61 | 31 | Atypical |  |
| #22 | 57 | 30 | Typical and Atypical | Anti-Social Personality Disorder |
| #23 | 56 | 23 | Typical | Anti-Social Personality Disorder, Affective symptoms |
| #24 | 58 | 25 | Atypical | Poly drug use, Alcohol |
| #25 | 19 | 3 | Typical | Obsessive Compulsive Disorder |
